# New copolymers as hosts of ribosomal RNA

**DOI:** 10.1186/s13065-019-0555-1

**Published:** 2019-03-23

**Authors:** Magali Hernández, Gerardo Leyva, Jonathan J. Magaña, Ariel Guzmán-Vargas, Carlos Felipe, Víctor Lara, Enrique Lima

**Affiliations:** 1 Laboratorio de Fisicoquímica y Reactividad de Superficies (LaFReS), Instituto de Investigaciones en Materiales, Universidad Nacional Autónoma de México, Circuito exterior s/n, Cd. Universitaria, Del. Coyoacán, CP 04510 Mexico City, CDMX Mexico; 2 Facultad de Química, Universidad Nacional Autónoma de México, Circuito exterior s/n, Cd. Universitaria, Del. Coyoacán, CP 04510 Mexico City, CDMX Mexico; 3 Departamento de Genética, Instituto Nacional de Rehabilitación, Calz. México Xochimilco No 289, CP 14389 Mexico City, CDMX Mexico; 4 Instituto Politécnico Nacional - ESIQIE, Avenida IPN UPALM Edificio 7, Zacatenco, 07738 Mexico City, CDMX Mexico; 5 Centro Interdisciplinario de Investigaciones y Estudios sobre Medio Ambiente y Desarrollo (CIIEMAD), Instituto Politécnico Nacional, Calle 30 de Junio de 1520 s/n, Barrio la Laguna Ticomán, 07340 Mexico City, CDMX Mexico; 6 Universidad Autónoma Metropolitana, Iztapalapa, Av. San Rafael Atlixco No. 186, Col. Vicentina, CP 09340 Mexico City, CDMX Mexico

## Abstract

Functionalized copolymers were synthesized and are proposed as hosts of RNA. The copolymers are based on carboxymethyl cellulose and poly-(ethylene glycol)-OH. These copolymers were functionalized with two amino acids, either lysine or histidine, through amide bond formation. The functionalized copolymer was then used to adsorb ribosomal RNA. The RNA loading was based on the nature of the amino acid functionalization of the copolymer. The array of RNA-copolymers was observed to be soft sphere-like, where the density of spheres was a function of the molecular weight of the carboxymethyl cellulose and the nature of the amino acid. Such RNA-copolymer systems are very sensitive to changes in pH.

## Introduction

Nanoparticles are already being developed as effective carriers of drugs and gene delivery to target regions of the body that were previously hard to access using traditional drug formulation methods. Through manipulation of their elemental composition, charge, size, and chemical functionalization, it may be possible to target particles to specific organs [1].

Gene therapy uses nucleic acids as a powerful tool to cure genetic deficiencies or diseases that currently have no cure. This includes a number of brain diseases (Alzheimer’s and Parkinson’s disease), viral infections and cancer [2, 3]. Therapeutic RNA (SiRNA, ribozymes, and mRNA) and DNA (plasmid DNA; oligonucleotides) delivery have been limited by a number of factors. Naked, single-stranded RNA, degraded by a nuclease, activates the immune system and is negatively charged to passively cross the cell membrane; it must be able to enter the cell and escape from endosomes [4, 5].

Complex DNA nanostructures can be functionalized with different biomolecules or nanomaterials, such as different nanowires, nanoparticles, organic molecules, peptides or proteins, to combine the properties of both DNA and nanomaterials for achieving the aimed functionality [6]. Nucleic acids require encapsulation, protection and stability in nanosized carriers by using viral or nonviral vectors that enable efficient intracellular delivery [6]. The use of nonviral vectors is gaining attention due to their low immunogenicity compared with that of viral vectors [7]. Various nonviral vectors, such as cationic polymers, including polylysine and polyamidoamine, are used to electrostatically balance the negatively charged RNA or DNA; however, excess cationic components cause adverse reactions, such as platelet aggregation and inflammatory reactions [8, 9]. Dendrimers, gold nanoparticles, quantum dots and methacrylate/methacrylamide polymers have also been proposed [10–12]. However, it is necessary to improve these materials and develop new materials to avoid gene delivery problems. Additionally, it is important to consider the protection of DNA, ease of fabrication, ability to target specific cell types, inexpensive synthesis, facile purification, stability, internalization, endolysosomal escape, efficient unpackaging, nontoxicity, and nonimmunogenicity [13].

Given their high degree of chemical flexibility, polymers are commonly used materials for nanoparticle-based delivery [14, 15]. Polysaccharides are used for pharmaceutical and biomedical applications due to their biocompatibility and nonimmunogenic properties. Currently, there is growing interest in applying these polymers for the development of nanomedicines [16]. In this sense, carboxymethyl cellulose sodium salt (CMC) is a derivate of cellulose used in food, cosmetics and pharmaceutical products due to its high biocompatibility, biodegradability and low immunogenicity [17]. Another advantage is the ease of chemical modification due to the availability of various functional groups on the glycosidic units (hydroxyls, carboxylic acids) [18].

Furthermore, a polymer coating with polyethylene glycol (PEG) provides protection from uptake by human monocytes. Surface modification of nanoparticles with PEG prolongs the circulation time of the nanoparticles, temporarily avoiding the mononuclear phagocyte system [19]. This polymer is amphiphilic and soluble in water as well as in many organic solvents. This polymer is nontoxic and is approved by the U.S. Food and Drug Administration for use in different pharmaceutical formulations, cosmetics and foods [20].

The nucleic acid nanocarriers are usually captured by the cells and internalized via an endocytic uptake mechanism [21]; they should preferably escape from the endosomes in the cell cytoplasm to avoid nucleic acid degradation and release the encapsulated or complexed nucleic acid. Several strategies have been explored to improve the escape from endosomes such as the incorporation of fusogenic agents. The incorporation of histidine and imidazole into cationic polymers, cationic lipids or peptides has led to nucleic acid delivery [22]. Lipids/peptides in liposomes and polyplexes, with the introduction of an ionizable group, are efficient systems to generate a proton sponge effect inside endosomes [23, 24]. pH-responsive compounds, such as amino acids, have been incorporated into nanocarriers (conjugated) to achieve efficient intracellular delivery of complexed nucleic acid through electrostatic interactions. In this study, copolymers of carboxymethyl cellulose-polyethylene glycol were prepared and functionalized with histidine and lysine for use as carriers of ribosomal RNA.

## Experimental procedures

Carboxymethyl cellulose [CEKOL 700 (7CMC), MW-270,000; CEKOL 30 (3CMC), MW-80,000; degree of substitution (DS) = 0.82] was obtained from CPkelco. Hydrochloric acid, acetonitrile, diethyl ether, methanol, poly(ethylene glycol) methyl ether (mPEG-OH, MW = 2000), 1-ethyl-3-(3-dimethylaminopropyl)-carbodiimide HCl (EDC HCl), *N*-hydroxysuccinimide (NHS), 4-dimethylaminopyridine (DMAP), phosphate-buffered saline (PBS), sodium hydroxide, l-lysine, and l-histidine monohydrochloride monohydrate were purchased from Sigma-Aldrich. Quant-iT RiboGreen RNA Reagent was purchased from Fisher Scientific, Inc.

### Materials

#### Copolymers of carboxymethyl cellulose-polyethylene glycol

**Scheme 1:**
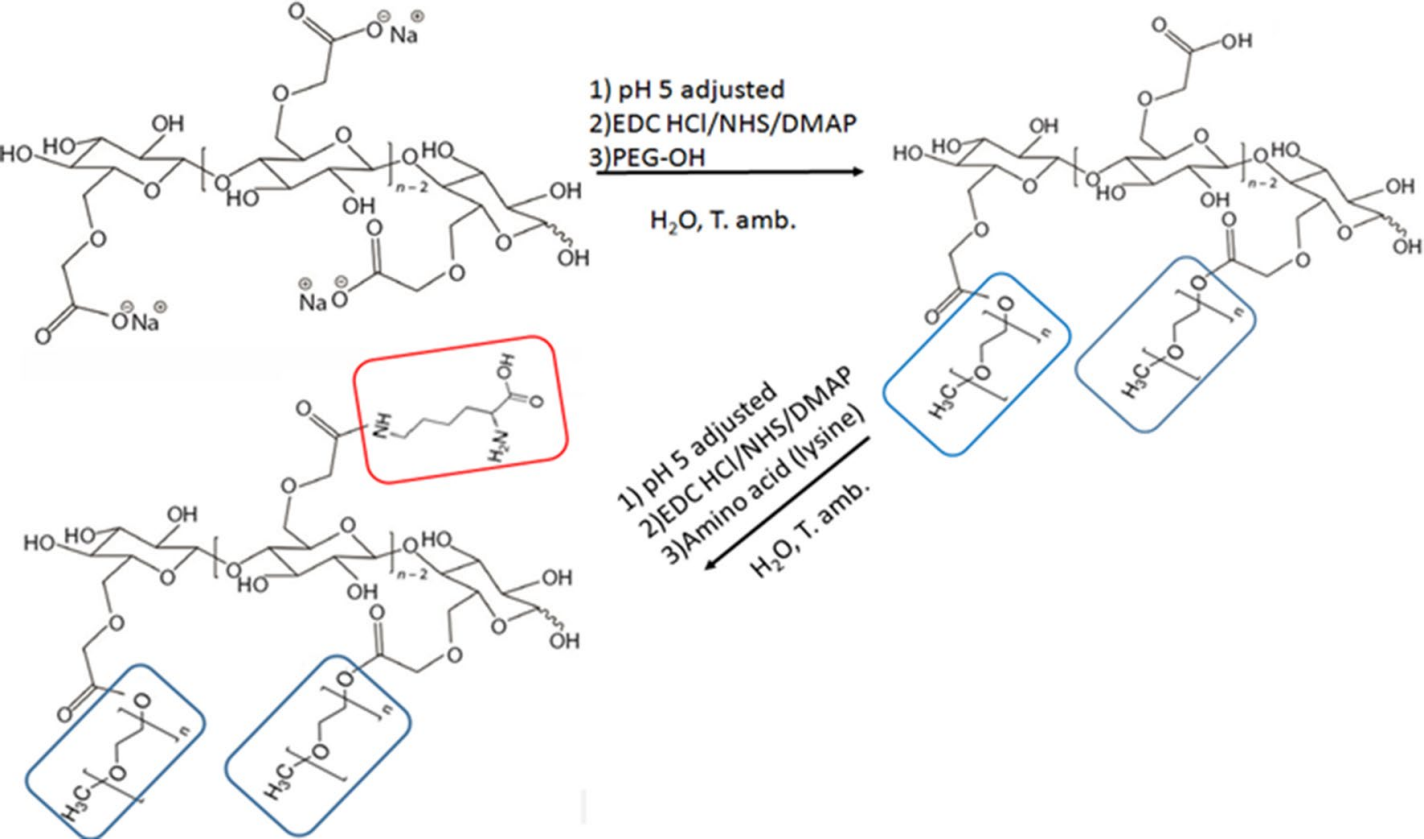
Schematic of functionalized copolymer preparation

Copolymers of carboxymethyl cellulose-polyethylene glycol were synthesized as follows: CMC (1.2 mmol acid) was dissolved in water (30 ml) and pH adjusted to 5 (HCl solution), to which EDC HCl (1.4 mmol), NHS (1.4 mmol), and DMAP (0.2 mmol) were added. The solution was stirred for 1 h at room temperature under dark conditions. After an hour, PEG-OH (0.36 mmol) reagent was added to the CMC-PEG solution. After overnight reaction, the product was precipitated with methanol and washed with a mixture of acetonitrile and diethyl ether (90:10). It was subsequently dissolved in water and precipitated in methanol, filtered and dried at room temperature. EDC HCl, DMAP, and NHS were dissolved in water (2 ml), and mPEG-OH was dissolved in water (4 ml). Two copolymers were synthesized and labeled 7CMC-PEG or 3CMC-PEG according to the precursor of CMC utilized in synthesis (Scheme 1).

#### Functionalization of copolymers with amino acids

Water at pH 5 was used to dissolve 7CMC-PEG or 3CMC-PEG. Then, EDC HCl (1.4 mmol), NHS (1.4 mmol), and DMAP (0.2 mmol) were added. Lastly, after 1 h, 0.36 mmol of amino acid, histidine or lysine was added (Scheme 1).

#### Preparation of nanoparticles of copolymers

The formulation, composed of copolymers (carboxymethyl cellulose-PEG-amino acid) and Tween 80 solution, was prepared by dissolving 3 mg of copolymer in 2.5 ml of sterile solution. The solution at 6% was prepared by diluting Tween 80 in sterile deionized water. The samples were sonicated for 2 h and centrifuged at 12,000 rpm for 30 min. The nanoparticles were suspended in sterile deionized water, and the size of the nanoparticles was measured by dynamic light scattering.

### Characterization

The structures of the materials were characterized by infrared (FTIR) and ^13^C nuclear magnetic resonance (CP MAS NMR) spectroscopies. FTIR spectra (ATR mode) of samples were acquired using a Bruker Alpha FTIR spectrometer. The spectra were recorded at a resolution of 4 cm^−1^ in the spectral window 4000–400 cm^−1^. ^13^C CP MAS NMR spectra were obtained at a Larmor frequency of 75.4 MHz using a Bruker Avance 300 spectrometer equipped with a 4 mm cross-polarization (CP) MAS probe. The samples were spun at a rate of 5 kHz. Spectra were recorded using a contact time of 5 ms and *π*/2 pulses of 5 µs. The chemical shifts were referenced to TMS.

The thermal properties of the materials were characterized by thermogravimetric analysis (TGA). TGA profiles were obtained using a TGA 500 system. Samples were heated at a heating rate of 10 °C/min from 30 to 900 °C under a nitrogen atmosphere with a flow rate of 1.0 ml/min.

The average hydrodynamic diameter, zeta potential and polydispersity index (PDI) of the nanoparticles were measured by dynamic light scattering (DLS) analysis using a Zeta sizer Nano ZS90 (Malvern Instruments). All measurements were carried out at 25 °C, at a detection angle of 90° (for size and PDI) and 120° for zeta potential.

### Ribosomal RNA onto copolymers

The nanoparticles were suspended in ribosomal RNA solution (2 ml with a 100 ng/ml concentration). The solution was incubated at 8 °C for 10 min. Then, the mixture was centrifuged at 12,000 rpm for 30 min to pellet the nanoparticles. The supernatant was removed and analyzed by the Quant-iT RiboGreen assay (Invitrogen). The amount of ribosomal RNA in the supernatant (w) was then subtracted from the total amount of ribosomal RNA added (w, 100 ng). The experiments were conducted in triplicate. The percentage efficiency (E) of ribosomal RNA entrapment to the nanoparticles was calculated using the following formula:

$${\text{E}} = \left( {{\text{total amount of ribosomal RNA }}\left( {\text{w}} \right) - {\text{free ribosomal RNA in supernatant }}\left( {\text{w}} \right)} \right)/\left( {{\text{total amount of ribosomal RNA }}\left( {\text{w}} \right)} \right) \times 100$$

Measurement data were expressed as the mean ± standard deviation; the comparison between two groups was analyzed using a two-sample t-test. Statistical analysis was performed using GraphPad Prism ver. 5.0 (GraphPad software, USA). P values ≤ 0.05 were considered statistically significant.

The nanoparticles loaded with ribosomal RNA were suspended in sterile phosphate-buffered saline (PBS) at a pH of 5, 6 and 7.4. The pH of the PBS was modified using 0.1 M NaOH or 0.1 M HCl. The mixture was then sonicated for 1 h. The nanoparticles were stored at 8 °C for periods as long as 1 week. The particle size and zeta potential of polymer complexes were measured at various time points (1 h, 24 h and 1 week).

### Array of components of hybrid copolymer-RNA

Small-angle X-ray scattering profiles were obtained using a Kratky camera coupled to a copper anode tube. The distance between the sample and the linear proportional counter was 25 cm; a Ni filter selected the Cu KR radiation. The sample was introduced into a capillary tube. The intensity I(q) was measured for 9 min to obtain high-quality statistics. The SAXS data were processed with the ITP program [25–27], where the scattering angle (q) is defined as q = 2πsinθ/λ, where θ and λ are the X-ray scattering angle and the wavelength, respectively. The shape and size distribution function of the scattering objects was estimated from the Kratky plot, q^2^I(q) against q. Lastly, the fractal dimension of the scattering objects was estimated from the slope of the curve log I(q) vs log(q) [28, 29].

## Results and discussion

### Functionalization of copolymers

**Fig. 1:**
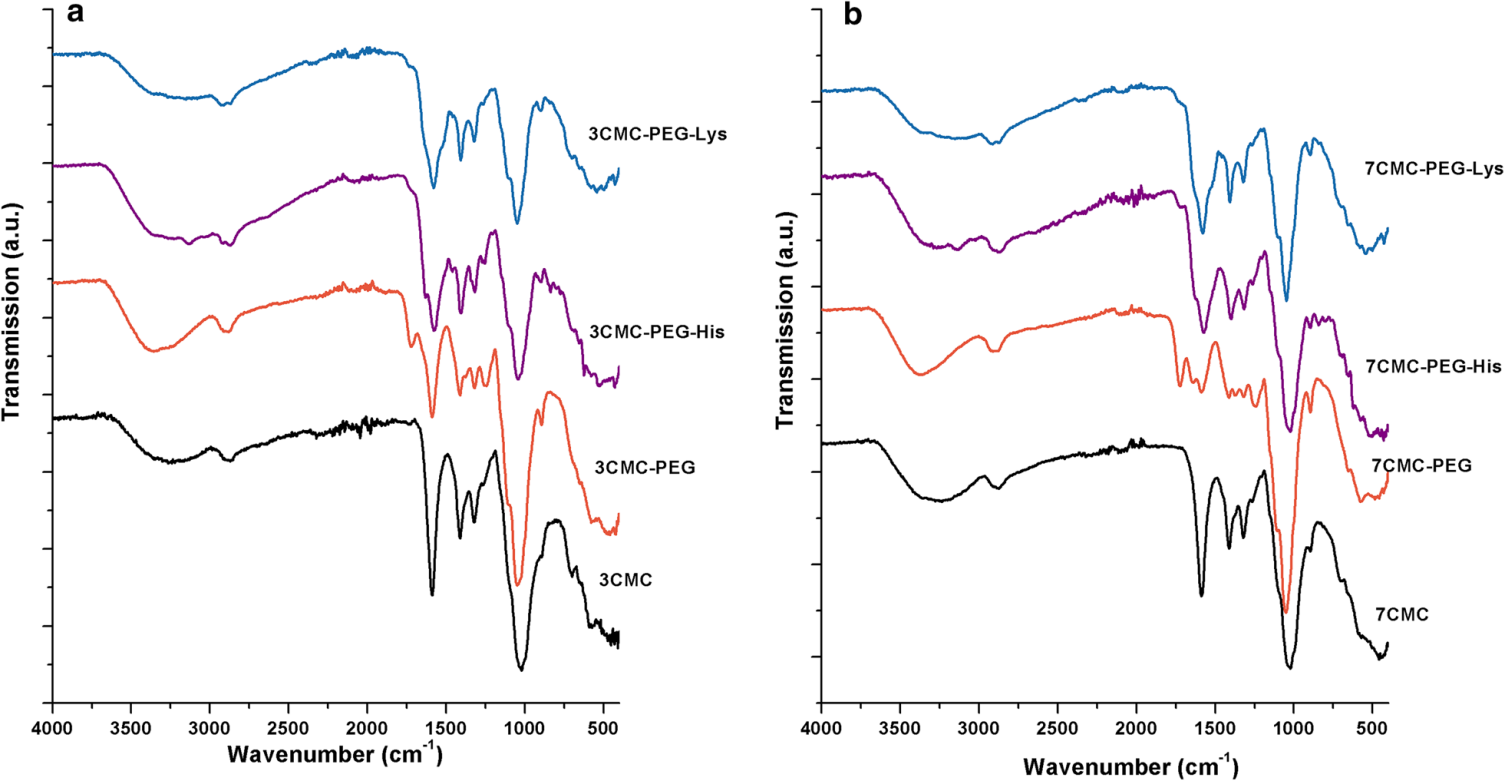
FTIR spectra of copolymers, **a** spectra of 3CMC and 3CMC functionalized with PEG and amino acids (lysine and histidine). **b** Spectra of 7CMC functionalized with PEG and amino acids

The FTIR spectra of two polymers functionalized with amino acids are shown in Fig. 1. The spectra of carboxymethyl celluloses are also included as a reference. The spectra of both celluloses, 3CMC and 7CMC, present a broad absorption band at 3422 cm^−1^, which is due to the stretching vibrational mode of the hydroxyl group. The band at 2924 cm^−1^ is assigned to the ν_C-H_ stretching vibration. The absorption band at 1613 cm^−1^ is assigned to ν C=O present in carboxyl group. The band at 1062 cm^−1^ is due to C–O–C stretching. The spectra of copolymers 3CMC-PEG and 7CMC-PEG fit relatively well with that of celluloses; however, it must be emphasized that the bands at 1740 cm^−1^ for 3CMC-PEG and at 1742 cm^−1^ for 7CMC-PEG are ascribed to the C=O stretching mode of the ester groups [30, 31]. These bands confirm that polyethylene glycol (PEGOH) had been linked to sodium carboxymethyl cellulose. The spectra of copolymers functionalized with histidine and lysine, 3CMC-PEG-His and 3CMC-PEG-Lys, did not show significant differences from that of 3CMC. A similar behavior was observed with the spectra of functionalized copolymers 7CMC. To obtain complementary information about the structure of these materials, solid-state ^13^C NMR measurements were performed.

**Fig. 2:**
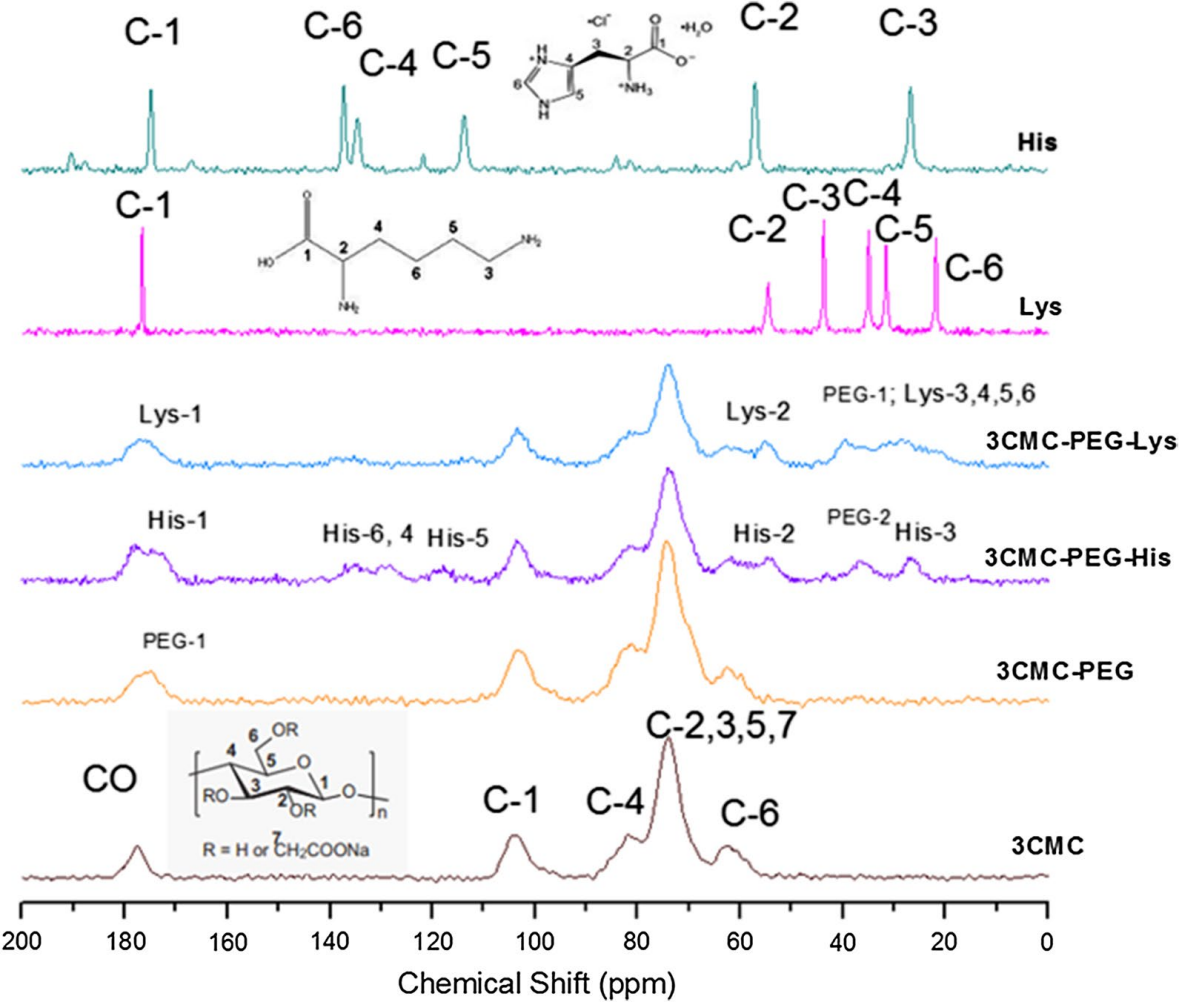
^13^C CP MAS NMR spectra of carboxymethylcellulose (low molecular weight, 3CMC) and carboxymethylcellulose functionalized with PEG and amino acids (lysine, histidine)

Figure 2 displays the ^13^C CP MAS NMR spectra of celluloses and amino acids as references and their corresponding copolymers. In the spectra of 3CMC and 7CMC, the broad NMR peaks at 178, 104, 83, 75, and 63 ppm were assigned to the carbonyl carbons, C1 and C4, and the overlap of C2, C3, C5, C7, and C6, respectively [32]. The peaks due to histidine were resolved at 174.94, 137.36, 134.42, 67.06 and 26.86 ppm. The assignment of these peaks is performed for the corresponding spectrum according to the number of carbons in Fig. 2.

The spectra of 3CMC-PEG and 7CMC-PEG exhibit a new signal close to 177 ppm, in addition to the resonances of celluloses. This peak suggests that the –COOH groups of carboxymethyl cellulose reacted with the hydroxyl groups of PEGOH to form the ester crosslink. In the spectrum of 3CMC-PEG-His, Fig. 2, the peaks between 110 and 140 ppm, assigned to aromatic carbons, are broader, and a downfield shift was observed for carbons C-2 (carbon bonded to amine in histidine). Furthermore, an additional peak at 174 ppm was observed. These observations can be explained as the aromatic ring of histidine interacting with the 3CMC-PEG surface and CMC-PEG reacting with the amino groups (C-2) of histidine to form the amide link. Regarding the spectra of copolymers containing lysine, the broadness of aliphatic carbons C3, C4, C5, and C6 indicates that lysine interacts with the copolymer through the amine at the end of chain, reducing the motion of four carbons.

**Fig. 3:**
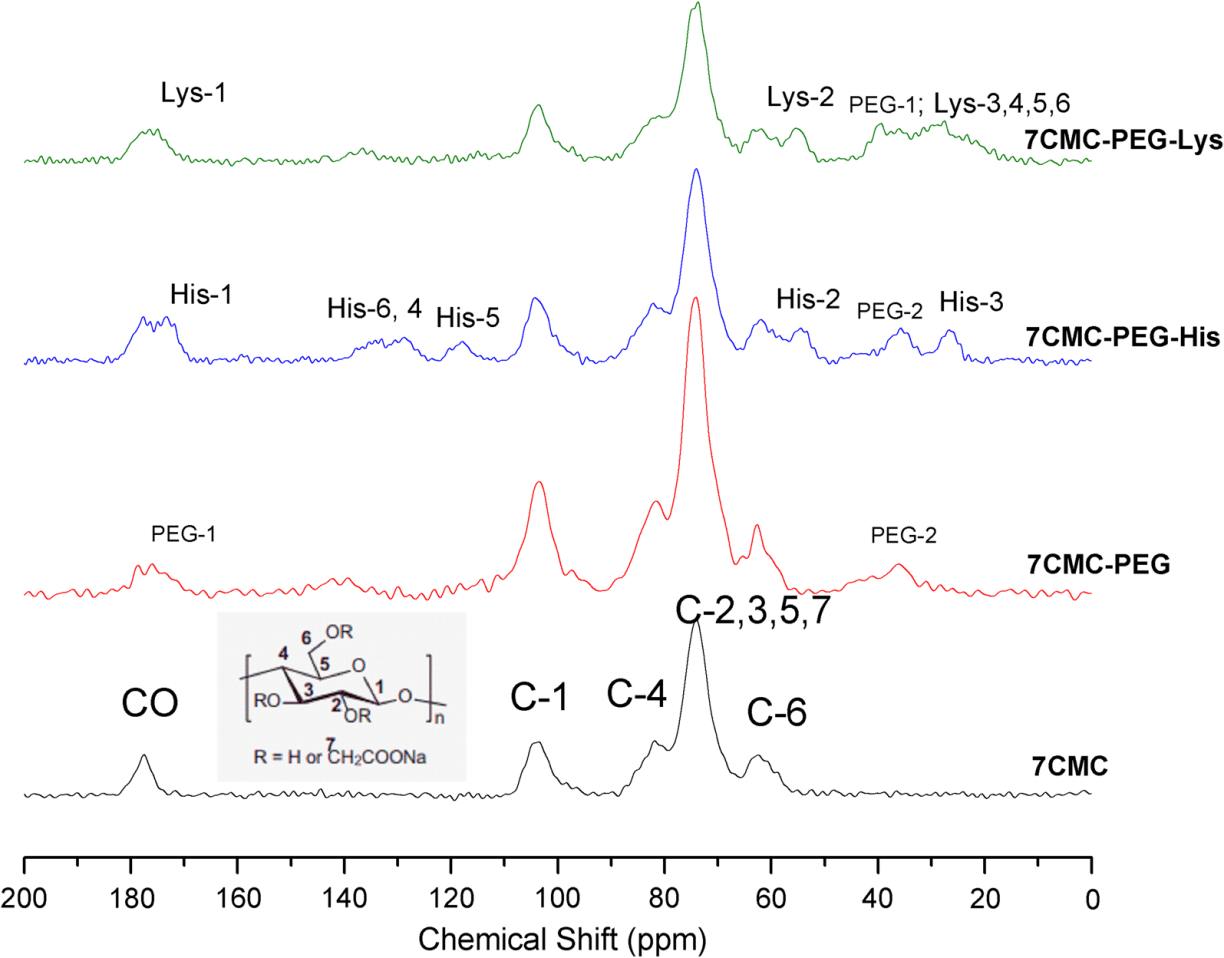
^13^C CP MAS NMR spectra of carboxymethylcellulose (high molecular weight, 7CMC) and carboxymethylcellulose functionalized with PEG and amino acids (lysine, histidine)

The ^13^C CP MAS NMR spectra in Fig. 3 correspond to samples synthesized with 7CMC, and a similar behavior to the series of samples with 3CMC was observed.

**Fig. 4:**
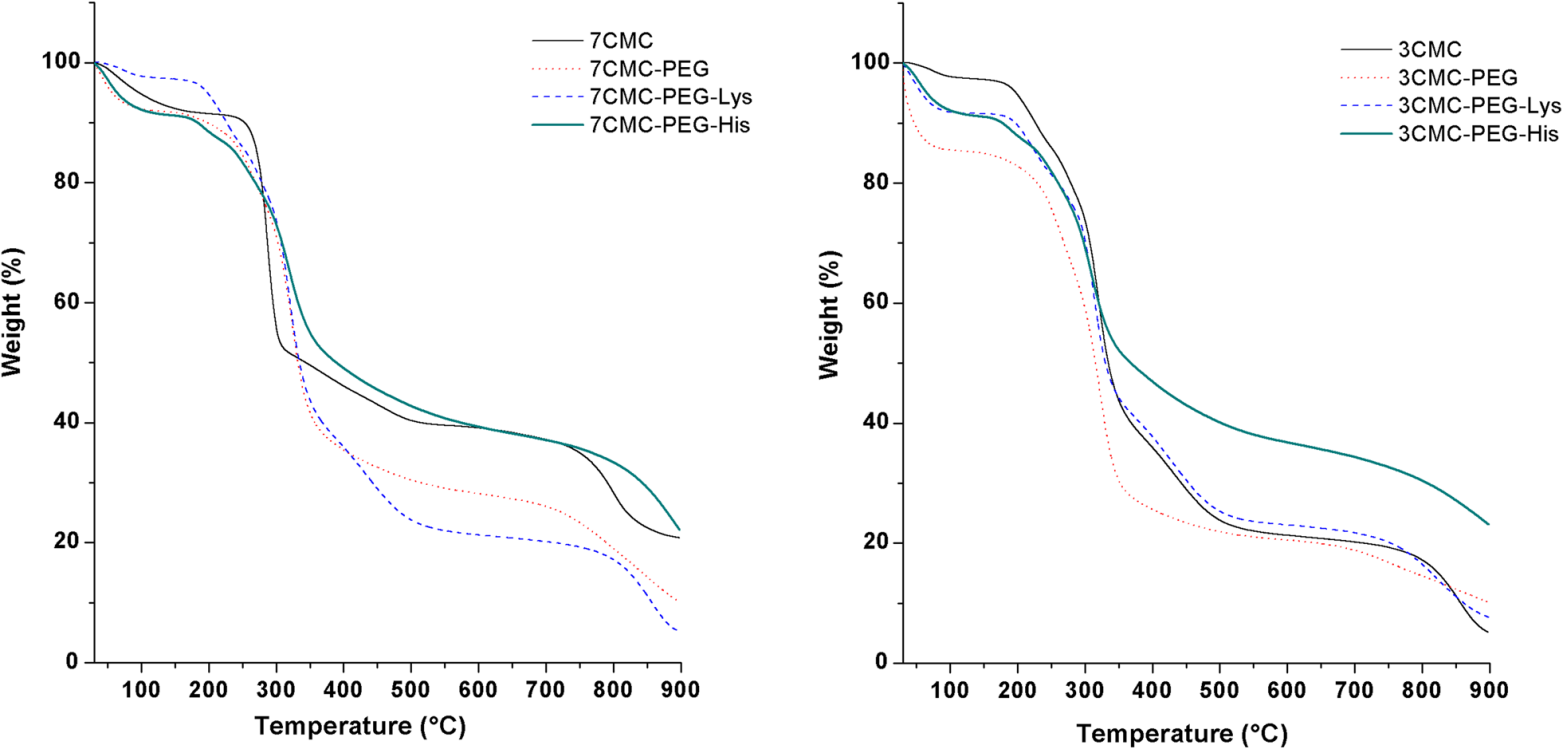
TGA curves of copolymers and polymers 7CMC (left), 3CMC (right)

The TG curves of copolymers prepared from 7CMC are shown in Fig. 4. The degradation of polymers occurs in two steps. The first TG step (2–15% mass loss), due to the loss of adsorbed water, occurs in the range from 30 to 150 °C. The second TG step occurs in the 250–460 °C temperature range, with a mass loss (30–40%). This step is attributed to the degradation of the side chain and the loss of CO_2_ [33, 34]. The copolymers began to degrade at approximately 150 °C, and the final decomposition temperature was found at approximately 450 °C. The higher decomposition temperature indicates that copolymers are more thermally stable than 7CMC and 3CMC. Table 1 compares the degradation temperatures and percentage mass for individual polymers and their blends. The TGA profiles of copolymers with PEG or amino acids show more than two degradation steps. The two mass losses between 150 and 260 can be attributed to the loss of CO_2_, –OCH_3_ groups of PEG and –NH_2_–R groups.

**Table 1:** TG data for carboxymethycellulose and copolymers

Sample	Decomposition step	Temperature (°C)		Weight loss %
		Start	End	
7CMC	1st	30	175	8
	2nd	175	350	42
7CMC-PEG	1st	30	150	8
	2nd	150	280	14
	3rd	280	420	43
7CMC-PEG-His	1st	30	150	8
	2nd	150	215	5
	3rd	215	270	10
	4th	270	450	30
7CMC-PEG-Lys	1st	30	150	2
	2nd	150	250	13
	3rd	250	280	9
	4th	280	400	40
3CMC	1st	30	150	2
	2nd	150	255	14
	3rd	250	380	40
3CMC-PEG	1st	30	150	15
	2nd	150	285	20
	3rd	285	450	40
3CMC-PEG-His	1st	30	150	8
	2nd	150	250	8
	3rd	250	382	39
3CMC-PEG-Lys	1st	30	150	8
	2nd	150	210	6
	3rd	210	270	8
	4th	270	460	34

### Particle size distribution

The data reported in Table 2 include the particle size, the polydispersity index (PDI) and the zeta-potential values. The particle size ranged between 9 and 14 nm. Particles of copolymers prepared with 7CMC were smaller than those prepared from 3CMC, suggesting that molecular weight is an important parameter influencing the crosslinking between cellulose and polyethylene glycol. Loading amino acids induces an increase in particle size; however, no direct correlation was observed between amino acids and increasing particle size. It should be mentioned that particles of copolymers containing 7CMC were the materials with the largest PDI values. In fact, a width of 30% PDI is often considered the frontier between mono- and polydisperse. This corresponds to PDI equal to 0.09, which leads to the recommendation that PDI < 0.1 is monodisperse and PDI > 0.1 is multimodal. Thus, all the prepared copolymers are multimodal, except 3CMC-PEG-Hys, and these results are in line with previous results obtained for other polymeric systems [35–37]. Lastly, a remarkable difference was observed between polymers containing 3CMC and 7CMC. When amino acids are loaded onto copolymers with 7CMC, the Z-potential becomes more positive with respect to that of amino acid-free copolymers. In contrast, the opposite trend was observed for the series containing 3CMC. This result can be explained as the degree of substitution between 7CMC and 3CMC polymeric chains being very different because the substitution groups drive the surface interactions, which in turn determine the electrostatic charge.

**Table 2:** Particle size (diameter), polydispersity index (PDI) and Zeta potential of samples under study as determined by DLS data

Sample	Without ribosomal RNA			Loaded with ribosomal RNA		
	Particle size ± SD (nm)	PDI	Zeta potential (mV)	Particle size (nm)	PDI	Zeta potential (mV)
7CMC-PEG	9.83 ± 0.12	0.151	− 25.3	12.98 ± 0.07	0.22	− 25.9
7CMC-PEGLys	12.24 ± 0.07	0.34	− 25.06	9.14 ± 0.11	0.16	− 17.6
7CMC-PEGHis	11.39 ± 0.075	0.25	− 24.66	11.27 ± 0.09	0.22	− 20.9
3CMC-PEG	11.2 ± 0.035	0.25	− 3.22	14.18 ± 0.05	0.27	− 27.6
3CMC-PEGLys	13.01 ± 0.039	0.099	− 5.95	11.92 ± 0.15	0.22	− 19.9
3CMC-PEGHis	13.9 ± 0.049	0.175	− 9.84	13.96 ± 0.02	0.23	− 23.4

### RNA as guest in CMC-PEG-amino acid hosts

**Fig. 5:**
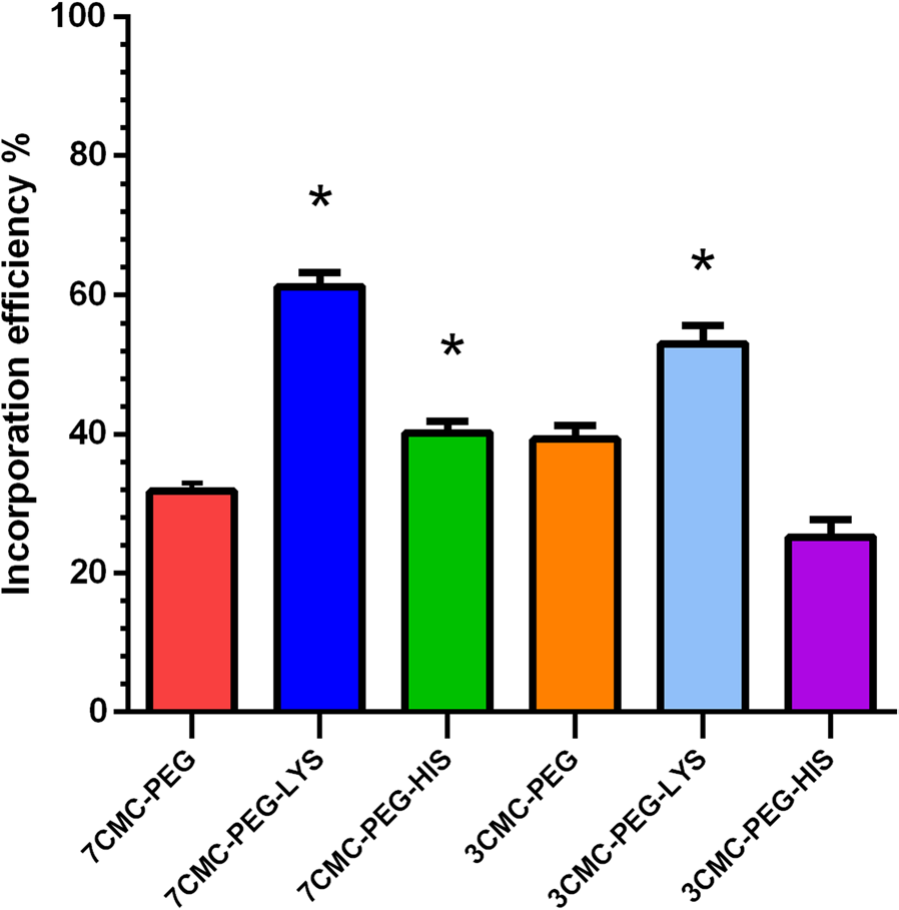
Ribosomal RNA (Maximal loading) on different copolymers. Experiments were performed by triplicate and standard deviation included in graphs

The histogram of Fig. 5 plots the amount of RNA adsorbed on different copolymers. The loaded RNA amount is expressed as a percentage of the RNA retained by copolymers from the total amount put in contact with particles. The highest fraction of RNA was loaded in both copolymers functionalized with lysine. The copolymer retaining the lowest fraction of RNA was 3CMC-PEG-His. The statistical parameters confirmed the efficiency of copolymers containing lysine. Compared to that in 7CMC-PEG, the incorporation efficiency in 7CMC-Lys was significantly increased (t = 21.94. P = 0.0001), as was the incorporation efficiency of 3 CMC-PEG LYS compared to that of 3 CMC PEG (t = 7.236, P = 0.0019).

**Fig. 6:**
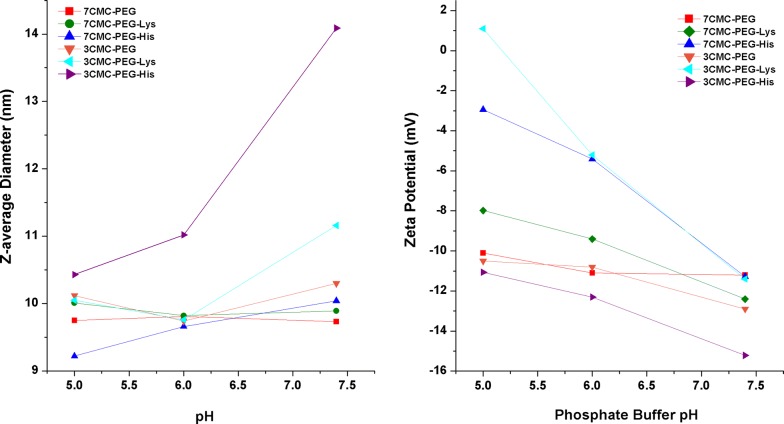
Size of particle and Zeta potential of copolymers as a function of pH variations. Copolymers were stored in phosphate-buffered solutions at pH 5.0, 6.0 and pH 7.4

**Scheme 2:**
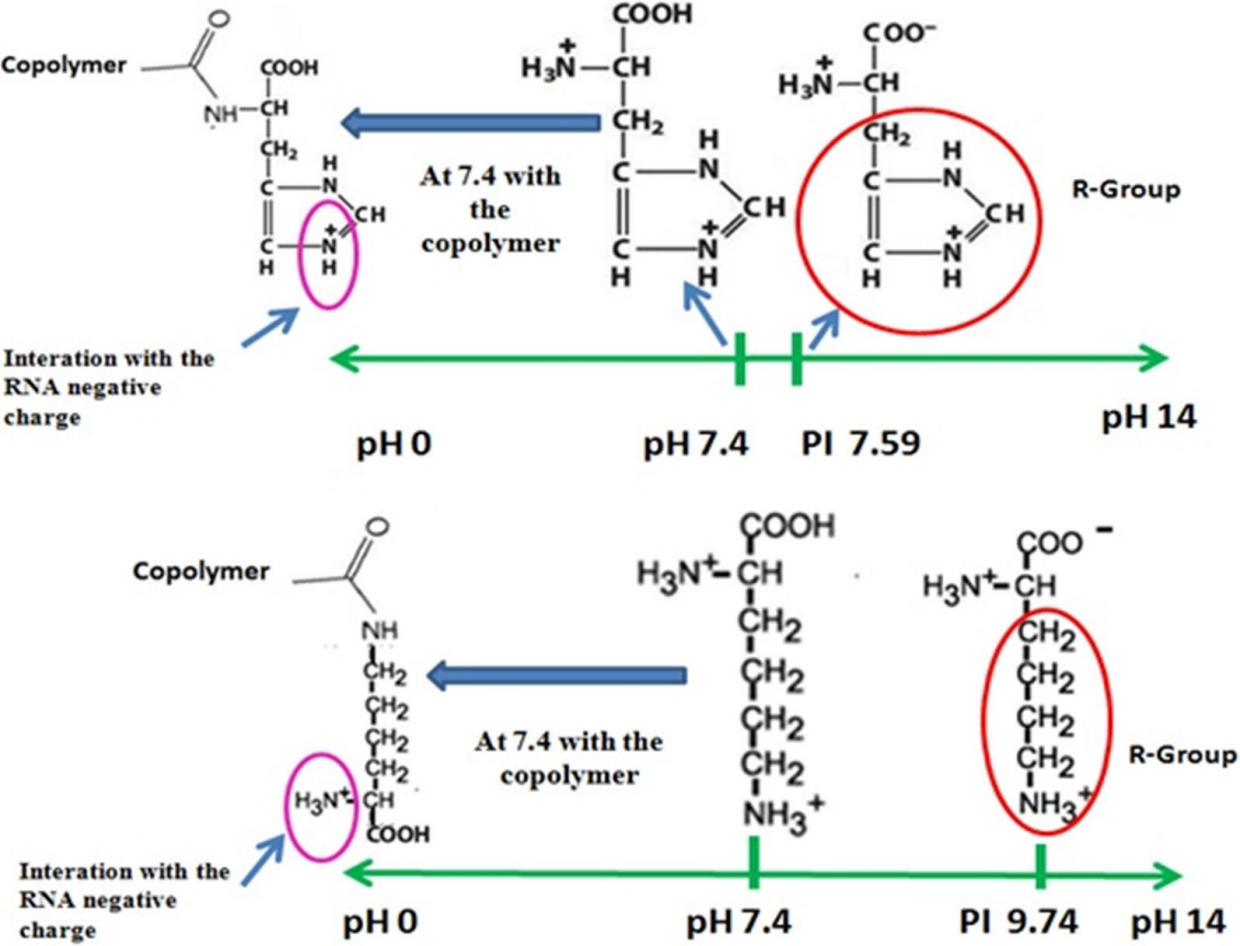
Isoelectric point of aminoacids anchored to copolymers. The carboxyl groups have a negative charge (− 1). But one-half of the amino groups and one-half of the R-Groups (–NH_3_^+^) have a positive charge, so the sum of their charge is + 1. At pH lower than IP, amine and carboxyl groups are protonated and at values higher than IP, ionizable groups donate protons

RNA loading, of course, changes the particle size, as shown in Table 2. In both amino acid-free copolymers, the particle size increases approximately 3 nm after RNA incorporation. The PDI value increases slightly upon RNA loading on both 7CMC-PEG and 3CMC-PEG copolymers. This result suggests that as a consequence of RNA loading, particles have a broader molecular weight distribution. This effect was observed earlier in polymeric systems where the RNA loading did not reach a saturation level [38]. The size does not increase with RNA adsorption onto all amino acid-copolymer systems. The copolymers functionalized with histidine had almost the same particle size before and after the RNA loading. The size of particles containing lysine decreases by approximately 2 nm, which is an unexpected result explained as follows: the acidic hydrogen permits a strong interaction between the functionalized surface of the copolymer and RNA. This compactness (or condensation) behavior was also previously observed in other polymeric systems [39, 40], and it will be confirmed below by SAXS results. Furthermore, the presence of RNA on particles significantly changes the Z-potential values. With RNA, the Z-potential becomes more positive for samples prepared from 7CMC and more negative for samples prepared from 3CMC. Before RNA loading, the Z-potential value was very different between samples containing 3CMC and that prepared from 7CMC. However, this difference was clearly lessened after RNA loading. Interestingly, the most positive materials containing RNA are those using lysine in functionalization, and the most negative are those free of amino acids. RNA is anchored to the surface of copolymers, but the surface properties of copolymers are crucial to the resulting hybrid RNA-copolymer materials. Because of these results, it is expected that materials differ significantly in response to changes in pH. The incorporation of pH-sensitive amino acids onto copolymers enables controlled endolysosomal escape. In this context, Fig. 6a plots the particle size of RNA-copolymers as a function of pH. The size of the nanoparticles decreases when the pH becomes acidic. At pH 7.4, the carboxyl groups are present in the form –COOH, and free amino groups (the other amino group forms the ester crosslink with CMC) are present in the protonated form, NH_3_^+^. Therefore, the electrostatic interaction between RNA and polymers becomes significantly favorable between the amino group and the negatively charged RNA, Scheme 2; consequently, the size of the particle decreases. No critical changes in the size of 7CMC-PEG and 3CMC-PEG were observed when the pH changed due to the absence of amino acids that give ionizable properties to the polymer.

To deduce the stability of particles, they were suspended in water at different pH values, and the particle size was measured over time. The results are summarized in Table 3. It is remarkable that at the same pH value, only slight changes were observed for the copolymers functionalized with amino acids, i.e., they were stable for periods as long as 1 week. At same time, the pH value is a parameter that also slightly influences the particle size, but no simple correlation was observed. The samples without amino acids, 7CMC-PEG and 3CMC-PEG, form agglomerates for periods as long as 24 h. It should be concluded that the incorporation of amino acids in the polymer chains generates stability in the aqueous medium.

**Table 3:** Particle size (diameter (nm)) of RNA-copolymers under different conditions of pH

Sample	Deionized water pH 7			pH 5			pH 6			pH 7.4		
	1 h	24 h	1 week	1 h	24 h	1 week	1 h	24 h	1 week	1 h	24 h	1 week
7CMC-PEG												
Size	12.98	29.09	50.72	9.75	9.49	9.74	9.81	11.72	120.86	9.73	11.51	10.32
PDI	0.22	0.2	0.38	0.05	0.1	0.16	0.09	0.28	0.34	0.06	0.24	0.16
7CMC-PEGLys												
Size	9.34	8.97	9.53	10.01	10.04	10.02	9.82	9.81	9.89	9.89	45.95	10.04
PDI	0.16	0.14	0.15	0.13	0.19	0.13	0.1	0.16	0.09	0.13	0.15	0.18
7CMC-PEGHis												
Size	11.27	11.94	13.71	9.22	9.78	9.88	9.66	9.75	9.98	10.04	9.96	10.09
PDI	0.22	0.15	0.32	0.12	0.09	0.1	0.07	0.11	0.13	0.08	0.16	0.14
3CMC-PEG												
Size	14.18	26.37	176.3	10.12	10.17	10.28	9.74	10.39	9.96	10.3	10.01	11.65
PDI	0.27	0.16	0.28	0.12	0.16	0.16	0.08	0.2	0.13	0.31	0.22	0.31
3CMC-PEGLys												
Size	11.92	12.12	12.31	10.05	11.52	9.67	9.76	9.89	10.54	11.16	119.1	59.75
PDI	0.22	0.24	0.22	0.08	0.22	0.06	0.07	0.09	0.15	0.12	0.23	0.18
3CMC-PEGHis												
Size	13.96	15.39	14.62	10.43	10.58	10.41	11.02	13.62	11.0	14.09	10.04	10.51
PDI	0.23	0.4	0.45	0.17	0.16	0.18	0.24	0.35	0.26	0.21	0.21	0.25

Interestingly, the particle size of the sample with lysine (7CMC-PEG-Lys) remained stable from pH 7.4 to acidic pH. This can be attributed to the value of the isoelectric point (pH where the molecule has a net charge of zero), in the case of lysine, which is 9.74. At pH 7.4, it is probable that the amino groups and the carboxyl group are completely protonated. The histidine has an isoelectric point of 7.59. At physiological pH, it is probable that not all the –COOH and NH_2_ groups of the copolymer are protonated, Scheme 2.

In Fig. 6, the zeta potential measured in different copolymers stored at different pH values is plotted. The polymers presented a negative charge under a physiological environment (pH 7.4), but the surface charge became slightly more positive at lower pH. The change in zeta potential can be attributed to protonation of the amine group under low-pH conditions. The surface charges of polymers play a role in cell uptake and blood stability. Generally, positively charged polymers promote cellular uptake due to the greater affinity for negatively charged cell membranes, but they tend to undergo rapid clearance from blood due to the strong interaction with the serum. Furthermore, negatively charged polymers can resist protein absorption, leading to a longer blood circulation time [41].

In summary, the results suggest that changes in the pH allow the polymer complexes to escape endosomal entrapment at acidic pH values (5 and 6). Thus, as a consequence of the protonation of carboxyl and amine groups, the polymers could reversibly convert surface charges in response to pH, facilitating endosome escape and cellular uptake. The pH dependence of the charge could be modulated by varying the molar ratio of carboxyl or amino groups.

### Array of the RNA-copolymer hybrid system

**Fig. 7:**
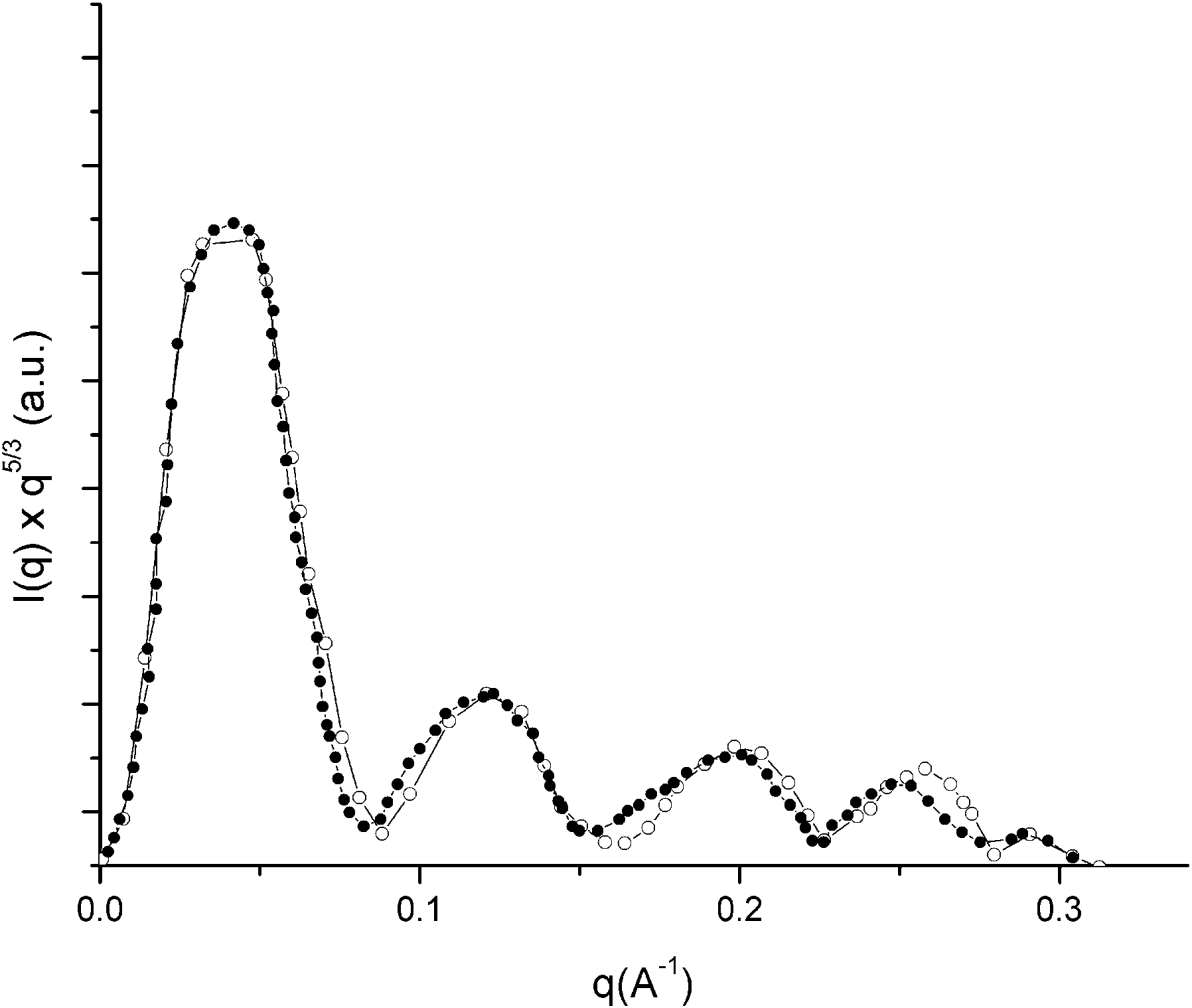
Modified Kratky profile for RNA loaded in 7CMC-PEGLys (empty circles) and, 7CMC-PEGHis (filled circles)

Figure 7 shows SAXS profiles, more precisely the Kratky modified plots [*I*(*q*) ×* q*^*5/3*^ vs *q*], of the RNA-copolymers. The modified Kratky plots have a main peak as well as a number of secondary peaks, and these profiles indicate that scattered objects have a globular shape. No significant differences concerning the shape of scattered particles were observed among the copolymers under study. The copolymer particle sizes were estimated from the SAXS data curves by fitting to a poly-disperse system of spheres. Table 4 shows that the size of RNA-copolymers ranges between 12.42 and 13.88 nm. The highest particle size was achieved when copolymers were prepared from 3CMC, which is in line with results obtained from DLS data. In general, RNA-copolymers functionalized with histidine are larger than those functionalized with lysine, confirming that protonation of amino acids is an important parameter for determining the interaction of copolymers with RNA. In this context, the fractal dimension value is a useful parameter because the loading of RNA onto copolymers without amino acids leads to spherical dense particles. However, the fractal dimension decreases when RNA is incorporated into the copolymers functionalized with amino acids, i.e., the particle size increases but the density decreases. Among the two functionalized amino acids, the polymers containing histidine lead to the most and least dense RNA copolymers; in other words, the spheres are not “hard” but respond to interactions of amino acids with copolymers and with RNA.

**Table 4:** Particle size and fractal dimension of RNA-copolymer particles as determined by SAXS data

Sample	Loaded with ribosomal RNA	
	Particle size (nm)	Fractal dimension
7CMC-PEG	12.42	2.81
7CMC-PEGLys	12.61	2.76
7CMC-PEGHis	12.97	2.62
3CMC-PEG	13.01	2.89
3CMC-PEGLys	13.14	2.73
3CMC-PEGHis	13.88	2.66

## Conclusion

PEGylated link polymers of carboxymethyl cellulose were effectively synthesized by EDC-NHS reaction and coupling amino acids onto the side groups of CMC-PEG copolymers. Copolymers were formed as stable soft spheres. Functionalization with lysine and histidine is crucial to obtaining copolymers prone to adsorbing ribosomal RNA; the type of amino acid determines the electric charge on the external surface of the spherical copolymer where RNA is incorporated. RNA-copolymers are partially stable as the amino acid-RNA interactions vary with pH, which could facilitate the release of RNA; this is a promising result because this RNA-copolymer system could then be used for RNA delivery.
